# Graded and Anisotropic Porous Materials for Broadband and Angular Maximal Acoustic Absorption

**DOI:** 10.3390/ma13204605

**Published:** 2020-10-16

**Authors:** Théo Cavalieri, Jean Boulvert, Gwénaël Gabard, Vicent Romero-García, Marie Escouflaire, Josselin Regnard, Jean-Philippe Groby

**Affiliations:** 1 Laboratoire d’Acoustique de l’Université du Mans, LAUM-UMR CNRS 6613, Le Mans Université, Avenue Olivier Messiaen, 72085 Le Mans CEDEX 9, France; jean.boulvert@univ-lemans.fr (J.B.); gwenael.gabard@univ-lemans.fr (G.G.); vicente.romero@univ-lemans.fr (V.R.-G.); jpgroby@univ-lemans.fr (J.-P.G.); 2 Safran Aircraft Engines, Rond Point René Ravaud - Réau, 77550 Moissy-Cramayel, France; marie.escouflaire@safrangroup.com (M.E.); josselin.regnard@safrangroup.com (J.R.); 3 Laboratoire d’Analyse Vibratoire et Acoustique, LAVA, Department of Mechanical Engineering, École Polytechnique de Montréal, P.O. Box 6079 Station Centre-ville, Montréal, QC H3C 3A7, Canada

**Keywords:** anisotropic materials, optimized absorption, diffuse field, graded properties

## Abstract

The design of graded and anisotropic materials has been of significant interest, especially for sound absorption purposes. Together with the rise of additive manufacturing techniques, new possibilities are emerging from engineered porous micro-structures. In this work, we present a theoretical and numerical study of graded and anisotropic porous materials, for optimal broadband and angular absorption. Through a parametric study, the effective acoustic and geometric parameters of homogenized anisotropic unit cells constitute a database in which the optimal anisotropic and graded material will be searched for. We develop an optimization technique based on the simplex method that is relying on this database. The concepts of average absorption and diffuse field absorption coefficients are introduced and used to maximize angular acoustic absorption. Numerical results present the optimized absorption of the designed anisotropic and graded porous materials for different acoustic targets. The designed materials have anisotropic and graded effective properties, which enhance its sound absorption capabilities. While the anisotropy largely enhances the diffuse field absorbing when optimized at a single frequency, graded properties appear to be crucial for optimal broadband diffuse field absorption.

## 1. Introduction

In the context of acoustic wave propagation, porous structures are commonly employed for sound absorption [1,2,3]. Their efficiency has been demonstrated many times over the past decades and they have been exploited in numerous applications, like civil engineering, room acoustics and building insulation. They are particularly useful for compact designs and cheap manufacturing. Their ability to absorb sound is often characterized by the diffuse field absorption coefficient which is defined as a weighted average of the absorption for all possible angles of incidence [4,5,6,7]. What would be considered as an optimal absorber would see its absorption coefficient maximized for all frequencies, under all possible angles of incidence. In order to envisage such an absorber, the design and optimization of anisotropic and graded materials are explored in this work.

Porous materials consist in two-phase media, in which a solid phase $\Omega_{s}$, here considered rigid and motionless is saturated by a fluid phase $\Omega_{f}$, in our case air [8]. The open porosity is denoted by the scalar $\phi =|\Omega_{f}|/|\Omega_{f}\cup \Omega_{s}|\in \left[0,1\right]$, and accounts for the ratio of the fluid volume over that of the total domain. In these conditions, the porous material can be treated as, whose acoustical properties depend on the micro-structure. Interestingly, as soon as the micro-structure is known, as for example in porous materials made of periodic arrangements of unit cells, the parameters of the Johnson–Champoux–Allard–Lafarge (JCAL) model can be efficiently computed by a two-scale asymptotic method. These parameters are the open porosity, high-frequency limit of tortuosity, characteristic thermal and viscous lengths, and static thermal and viscous permeabilities. The high-frequency limit of tortuosity can be interpreted as the path of a particle, in the inertial regime of the fluid. Both thermal and viscous characteristic lengths are related to thermal and viscous skin depth, and the size of the pores. Finally, the static viscous and thermal permeabilities are linked to the flow restitivty and compressibility. In the case of non-isotropic materials, the tortuosity, the characteristic viscous length and the static viscous permeability admit different values in each direction. The definition of these coefficients and their physical interpretation is detailed in Ref. [9]. The thermal and viscous dynamic regimes, occurring inside the medium, arise from the fluid-structure interaction at the microscopic scale of the unit cell [10]. The JCAL model uses frequency asymptotics to estimate these complex and frequency-dependent effective permeabilities of the porous medium described by the six parameters. In the most general case, the unit cells can be anisotropic and thus, the equivalent fluid can display anisotropic features. In this case, the mass density of the effective medium will be denoted by the symmetric, second-order tensor $\rho_{\left(e\right)}$ which accounts for in-plane and normal direction properties, together with the bulk modulus $B_{\left(e\right)}$. Such physical modeling has been demonstrated and validated experimentally in many instances [11,12]. The method consists in the application of a two-scale asymptotic homogenization to governing fundamental equations. The JCAL parameters are then calculated by integrating the computed fields [13,14,15].

In addition, the effective properties describing the wave propagation in the medium can also vary along a specific direction, specifically, together with the complex and frequency dependence, the medium displays spatially dependent features. Therefore, altering the micro-structure’s geometry at different locations affects the propagation of acoustic waves. In a rigorous manner, the solution of an anisotropic, spatially dependent Helmholtz equation describes the acoustic propagation inside the porous material. In this regard, various analytical and numerical methods of acoustic wave propagation have been proposed, for multi-layered and continuously space varying materials. Wave-splitting techniques with Green’s functions (WSTGF) or Peano series expansions (PS) can be applied for this purpose [16,17,18,19,20,21,22]. In order to reduce the computational cost, the well-known transfer matrix method (TMM) has been also used [23,24].

Recent work on optimized porous materials has been devoted to two main goals. On the one hand, maximizing sound absorption [14,22], and on the other hand recovering the JCAL parameters by developing inverse characterization methods [13]. It has been recently shown that quasi-perfect broadband absorption can be achieved at normal incidence, using graded porous layers [14]. In this work we present the optimization of anisotropic and graded materials for oblique incidences, paying special attention to the omnidirectional and broadband absorption. This relates to the property of maximal and uniform absorption for all possible angles of incidence [25]. Within the frame of this work, we use a set of unit cells which are described by their geometric parameters. In order to model the propagation in such materials, a database of JCAL parameters is established by means of finite elements method (FEM), as in Refs. [13,14,15]. We use a database of 100 anisotropic unit cells, which links the JCAL parameters to the geometric ones, for every unit cell. Starting from this set of homogenized anisotropic unit cells, variations of the geometry are introduced along the depth of the equivalent fluid layer, so as to achieve prescribed targets for the frequency and angular absorption. These targets are defined through the introduction of cost functions that are minimized based on the average and diffuse field absorption coefficients.

The present article is organized as follows: we first recall the general derivation of wave propagation in anisotropic and graded fluid layers in Section 2. We then discuss the interpretation of average and diffuse field absorption in Section 3. In Section 4 the data generation and the optimization procedures are described in detail. Finally, multiple numerical results are presented and discussed in Section 5 and are compared to those of isotropic and non-graded media, to demonstrate the benefits of anisotropic and graded materials.

## 2. Reminder on Anisotropic and Graded Porous Materials

**Figure 1:**
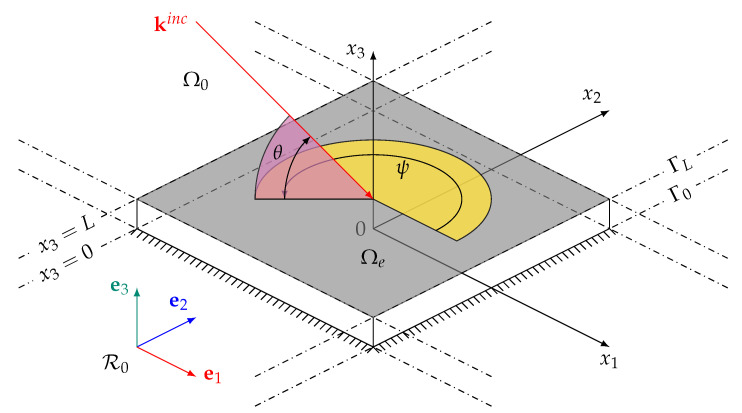
(Color online) Schematic of the propagation problem. The incident wave-vector $k^{inc}$ has azimuth and elevation angles ψ (yellow) and θ (light purple) respectively.

In this section the propagation of a plane wave through an anisotropic, uni-dimensional (1D) graded equivalent fluid is recalled. More in-depth knowledge and overall derivation of the equations can be found in the Refs. [13,14,22]. We set the Cartesian coordinate system $R_{0}=\left(O,e_{1},e_{2},e_{3}\right)$ with the associated spatial coordinates vector $x=\left(x_{1},x_{2},x_{3}\right)\in R^{3}$ as defined in Figure 1. The equivalent fluid domain, denoted $\Omega_{e}\equiv \Omega_{f}\cup \Omega_{s}$, is a slab of finite thickness *L* in the $x_{3}$ direction and of infinite extent in the $\left(O,x_{⊥}\right)$ plane. The subscript ${}_{⊥}$ denotes the restriction of a vector or tensor to the $\left(O,x_{⊥}\right)$ plane with $x_{⊥}=\left\{x_{1},x_{2}\right\}$. The domain $\Omega_{e}$ is bounded by the plane boundaries at $x_{3}=0$ and $x_{3}=L$ denoted $\Gamma_{0}$ and $\Gamma_{L}$ respectively. We solve for the sound field in this layer $\Omega_{e}$ in the linear harmonic regime using the time convention $e^{-i\;\omega t}$ where ω=2πf is the angular frequency. The exterior of the domain $\Omega_{e}$ is denoted $\Omega_{0}$ and corresponds to $x_{3}>L$. It contains an homogeneous isotropic fluid, taken to be air in this case and considered inviscid. The density of air is $\rho_{\left(0\right)}=1.213$ kg·m ${}^{-3}$ and its bulk modulus $B_{\left(0\right)}=\gamma P_{0}$ with γ=1.4 the ratio of specific heat and $P_{0}$ = 101,325 Pa the atmospheric pressure.

The pressure *p* and velocity **v** induced by the acoustic field are governed by the following linear equations for mass conservation and momentum conservation respectively,

(1a) $$\begin{array}{c} i\;\omega \rho_{\left(j\right)}\left(x_{3},\omega \right)v\left(x,\omega \right)=\nabla p\left(x,\omega \right)\;, \end{array}$$

(1b) $$\begin{array}{c} i\;\omega p\left(x,\omega \right)=B_{\left(j\right)}\left(x_{3},\omega \right)\nabla \cdot v\left(x,\omega \right)\;, \end{array}$$

where the subscript j={0,e} designates the domains $\Omega_{0}$ and $\Omega_{e}$ respectively. The effective bulk modulus and mass density tensor of the anisotropic 1D graded equivalent fluid (along the $e_{3}$ direction) are denoted $B_{\left(e\right)}\left(x_{3},\omega \right)$ and $\rho_{\left(e\right)}\left(x_{3},\omega \right)$. In the particular case of a transverse isotropic material, the density tensor can be written $\rho_{\left(e\right)}=\mathrm{diag}\left(\rho_{⊥},\rho_{⊥},\rho_{3}\right)$ in its principal directions. Note that these quantities are complex-valued, frequency-dependent and can vary along the $e_{3}$ direction. Moreover, while the bulk modulus of the anisotropic medium is scalar, the mass density is a second-order tensor accounting for the anisotropy. To summarize, a non-graded anisotropic material is modeled by the physical quantities $\rho_{\left(e\right)}\left(\omega \right)$ and $B_{\left(e\right)}\left(\omega \right)$, whereas for graded anisotropic materials, these quantities $\rho_{\left(e\right)}\left(x_{3},\omega \right)$ and $B_{\left(e\right)}\left(x_{3},\omega \right)$ are spatially dependent.

In the air region $\Omega_{0}$ ($x_{3}\geq L$) we define an incident plane wave with unit amplitude:

(2) $$p^{inc}\left(x,\omega \right)=e^{i\;k_{⊥}\cdot x_{⊥}-i\;k_{3}\left(x_{3}-L\right)}\;,$$

where the components of the wave-vector $k^{inc}={\left\{k_{1},k_{2},k_{3}\right\}}^{T}$ (with ${}^{T}$ the non-conjugate transpose) are given by

(3) $$\left\{\begin{array}{c} k_{1}=-k_{0}\cos\left(\theta \right)\cos\left(\psi \right),\\ k_{2}=-k_{0}\cos\left(\theta \right)\sin\left(\psi \right),\\ k_{3}=k_{0}\sin\left(\theta \right), \end{array}\right.$$

with ψ and θ the polar and elevation angles, respectively. The free-field acoustic wave-number is $k_{0}=\left|k^{inc}\right|=\omega /c_{0}$, and the sound speed in free air is defined by $c_{0}^{2}=B_{\left(0\right)}/\rho_{\left(0\right)}$.

The presence of the anisotropic layer $\Omega_{e}$ gives rise to a reflected wave $p^{r}$ in $\Omega_{0}$. It is written

(4) $$p^{r}\left(x,\omega \right)=Re^{i\;k_{⊥}\cdot x_{⊥}+i\;k_{3}\left(x_{3}-L\right)}\;,$$

where R(ω,θ,ψ) is the specular reflection coefficient and $k_{⊥}^{inc}={\left\{k_{1},k_{2}\right\}}^{T}$. The derivation of the governing equations Equation (1) has recently been used for retrieval techniques and applied to fully-anisotropic porous materials [13]. They can be written using a state-vector formulation,

(5) $$\frac{dW}{dx_{3}}=A\left(x_{3}\right)W\;,$$

where we have introduced the state vector $W={\left\{p,v_{3}\right\}}^{T}$, and the matrix

(6) $$A\left(x_{3}\right)=\left[\begin{array}{cc} 0 & i\;\omega \rho_{3}\\ i\;\omega B_{eq}^{-1} & 0 \end{array}\right]\;,$$

which admits no diagonal terms when the principal directions of the fluid are aligned to those of the coordinate system. The propagation problem is reduced to a system of equations with respect to $x_{3}$. The term $B_{eq}$ is the equivalent bulk modulus and accounts for transverse $\left(O,x_{⊥}\right)$ effective density and oblique incidence. In the case of anisotropic materials, with mass density tensor $\rho_{\left(e\right)}=H^{-1},H\in C^{3\times 3}$, bulk modulus $B_{\left(e\right)}$ and wave-vector k, we have,

(7) $$B_{eq}^{-1}=B_{\left(e\right)}^{-1}-\left[k_{⊥}\cdot \left(H_{⊥}\cdot k_{⊥}\right)\right]/\omega^{2}\;.$$

At the interface $\Gamma_{L}$ between the anisotropic layer and the surrounding fluid, the continuity of pressure and normal velocity is imposed as boundary conditions. At the interface $\Gamma_{0}$, a rigid backing is considered in this work, and thus zero normal particle velocity is imposed by ${v\cdot n|}_{\Gamma_{0}}=0$. As a consequence, the state-vector at both interfaces reads,

(8) $$W_{L}=\left\{\begin{array}{c} 1+R\\ \left(R-1\right)/Z_{0}^{\theta } \end{array}\right\}\;\mathrm{and}\;W_{0}=\left\{\begin{array}{c} p(0)\\ 0 \end{array}\right\}\;,$$

with $Z_{0}^{\theta }=Z_{0}/\sin\left(\theta \right)$ the apparent impedance of the air in domain $\Omega_{0}$ with respect to the unit outward normal vector $n=e_{3}$ at interface $\Gamma_{L}$. The system of equations in Equation (5) can be solved by various techniques, being the TMM, PS and WSTGF as in Refs. [16,17,18,19,20,21,22,23,24]. This way, we retrieve the reflection coefficient of graded and anisotropic porous materials with a rigid backing. The absorption coefficient is defined with respect to the angular frequency, and angles of incidence as

(9) $$\alpha \left(\omega ,\theta ,\psi \right)=1-{\left|R\left(\omega ,\theta ,\psi \right)\right|}^{2}\;.$$

To sum up, from a given geometry of the unit cell, one can compute the JCAL parameters as in Refs. [13,14,15] using FEM. From the JCAL parameters, we obtain the mass density tensor $\rho_{\left(e\right)}\left(\omega \right)$ and bulk modulus $B_{\left(e\right)}\left(\omega \right)$ of the homogenized medium [9]. As these effective properties depend on the geometry of the porous material, we can introduce variations of the geometric properties along the direction $x_{3}$ by using $\rho_{\left(e\right)}\left(x_{3},\omega \right)$ and $B_{\left(e\right)}\left(x_{3},\omega \right)$. Then, the reflection coefficient is provided by solving Equation (5), using the aforementioned numerical techniques.

## 3. Average and Diffuse Field Absorptions

**Figure 2:**
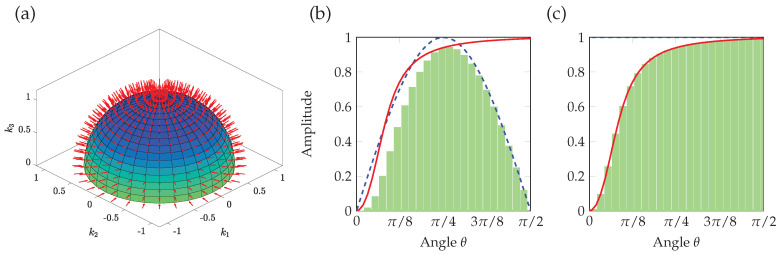
(Color online) Weighting process for average and diffuse field absorption coefficient. (**a**) Discrete incidences on the unit sphere and elementary surfaces. The subfigures (**b**,**c**) represent the integration process defined in Equations (11) and (12) respectively. The solid red line is an example of an angular-dependent absorption coefficient, the weighting function is shown in a dashed blue and the light green bars illustrate the discrete integration.

In the Cartesian coordinate system $R_{0}=\left(O,e_{1},e_{2},e_{3}\right)$, the incident wave is represented by its wave-vector $k^{inc}={\left\{k_{1},k_{2},k_{3}\right\}}^{T}$. The diffuse sound field is composed of a continuum of plane waves evenly distributed over the elevation angle θ and azimuth angle ψ. These plane waves have the same acoustic intensity and are uncorrelated [4,6,7]. To define an absorption coefficient for this diffuse field, the absorption coefficient α(ω,θ,ψ) defined above, in Equation (9) for an individual plane wave has to be averaged over all possible incidence angles. In this work, two distinct cases will be considered regarding this averaging of the acoustic absorption coefficient. The first average is based on the standard definition of the diffuse field absorption coefficient, which reads as follows [5],

(10) $$\alpha_{dif}\left(\omega \right):=\frac{\int_{0}^{\pi /2}\;\;\;\int_{0}^{2\pi }\alpha \left(\omega ,\theta ,\psi \right)\sin\left(\theta \right)\cos\left(\theta \right)d\psi d\theta }{\int_{0}^{\pi /2}\;\;\;\int_{0}^{2\pi }\sin\left(\theta \right)\cos\left(\theta \right)d\psi d\theta }\;,$$

where sin(θ)dθdψ is due to the change in area of the surface integration element on the sphere. In addition, the component of the acoustic intensity vector pointing into the surface is proportional to cos(θ). It follows that the normal and grazing angles of incidence are do not contribute in this definition, as illustrated in Figure 2b by the blue dashed line. The integral formalism in Equation (10) can be approached by a discrete sum. As described earlier in Section 2, we use transverse isotropic materials. Hence the polar angle ψ has no influence on the absorption, we now have,

(11) $$\alpha_{dif}\left(f\right)\approx \left(\sum_{i=1}^{N_{\theta }}\alpha \left(f,\theta_{i}\right)\sin\left(\theta_{i}\right)\cos\left(\theta_{i}\right)\right){\left(\sum_{i=1}^{N_{\theta }}\sin\left(\theta_{i}\right)\cos\left(\theta_{i}\right)\right)}^{-1}\;,$$

where $N_{\theta }$ is the number of elevation angles. The second average is based on the arithmetic average of the absorption coefficient, illustrated in Figure 2c:

(12) $$\bar{\alpha }\left(f\right)\approx \frac{1}{N_{\theta }}\sum_{i=1}^{N_{\theta }}\alpha \left(f,\theta_{i}\right)\;.$$

We note that when compared to the diffuse field average Equation (10), the angular weighting related to the variations of the solid angle and normal acoustic intensity are not accounted for in Equation (12).

## 4. Optimization Procedures

The optimization routines provide the anisotropic and graded properties of the porous layer for a given acoustic target. In particular, for a fixed layer thickness *L*, the macro-modulated effective density $\rho_{\left(e\right)}$ and bulk modulus $B_{\left(e\right)}$ are obtained for the given acoustic absorption properties.

### 4.1. Interpolated Database of Unit Cells

**Figure 3:**
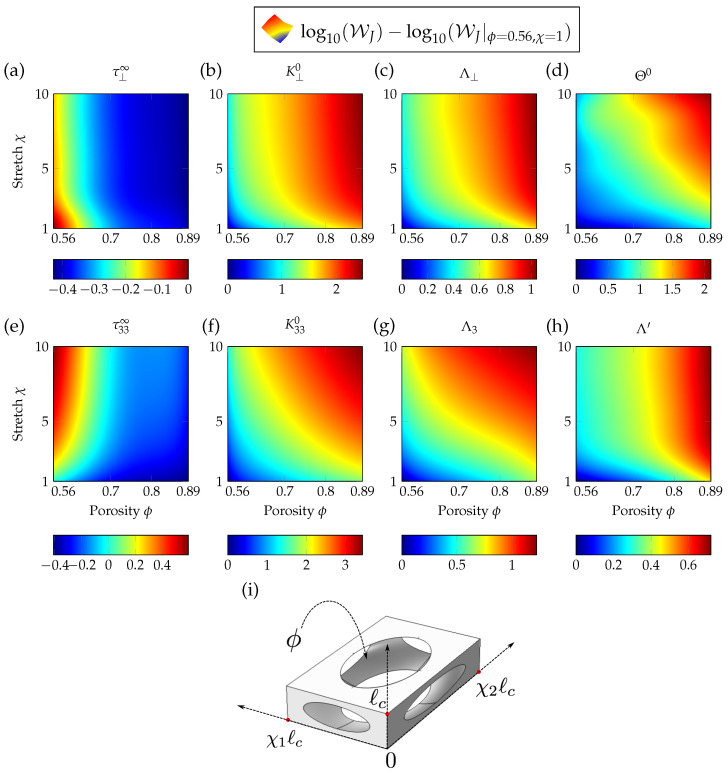
(Color online) Interpolated database of normalized JCAL parameters, with respect to the geometric properties ϕ and χ. The values are normalized to those of the isotropic cell with lowest porosity (corresponding to ϕ=0.56, $\chi_{1}=\chi_{2}=\chi =1$) and are displayed in a logarithmic scale. The values in the transverse direction of the tortuosity, static viscous permeability and characteristic viscous length are shown respectively in (**a**–**c**); the normal components are displayed in (**e**–**g**). Finally, the static thermal permeability and characteristic thermal length are given in (**d**,**h**) respectively. The unit cell is displayed along with its geometric assets in (**i**).

The proposed unit cell is made of an ellipsoid carved out from a rectangular cuboid, as illustrated in Figure 3i. This unit cell has already been used for retrieval methods in Ref. [13]. To define this geometry, several parameters are introduced, namely the open porosity ϕ, characteristic length $\ell_{c}$ and stretching in each in-plane direction $\chi_{1}$ and $\chi_{2}$. For simplicity, the two stretching parameters are set to be equal: $\chi_{1}=\chi_{2}=\chi$. A set of 100 unit cells with $\phi \in \left[0.56,0.89\right]$, and $\chi \in \left[1,10\right]$ is first used to build up a database of the JCAL parameters, representing 100 anisotropic porous materials made of the periodic repetition of the unit cell, with values $\left(\phi ,\chi_{1},\chi_{2}\right)$ from the previous intervals. The interval for porosity is driven by the topology of the unit cell, whereas the stretch is chosen to span one order of magnitude. These properties are obtained through a homogeneization method relying on finite elements methods (see [13,14] for more details). The graded anisotropic materials used in this work for the optimization of the acoustic absorption properties can be composed of layers of different materials, the values of porosity and stretching of each layer might correspond to a periodic porous material made of one of the unit cells in the given database.

In the case in which the couple (ϕ,χ) does not correspond to one of the 100 unit cells, the effective properties are obtained by interpolating between the data points existing in the database of JCAL parameters. This is done using piecewise cubic Hermite interpolating polynomials (PCHIP) [26]. The cubic interpolant of the transport parameters with the geometric parameters is monotonic. The use of such an interpolation method in this context is therefore robust. Figure 3 shows the dependency of the JCAL parameters on the stretching factor χ and open porosity ϕ. This interpolation spans the whole database and gives a set of JCAL parameters for the required unit cell, allowing us to obtain the corresponding mass density tensor and bulk modulus of the anisotropic porous material. We denote the vector of geometric parameters by $W_{G}={\left\{\ell_{c},\phi ,\chi \right\}}^{T}$ with which the unit cell is described. The corresponding JCAL parameters are stored in the vector of porous properties $W_{J}={\left\{\phi ,\Theta^{0},\Lambda^{\prime },\Lambda_{⊥},\Lambda_{3},\tau_{33}^{\infty },\tau_{⊥}^{\infty },K_{⊥}^{0},K_{33}^{0}\right\}}^{T}$.

The transport parameters shown in Figure 3, are computed for a unit characteristic length $\ell_{c}=1$ m. The parameters are then scaled independently according to their dimension, in meters for $\Lambda^{\prime }$, $\Lambda_{⊥}$ and $\Lambda_{3}$, in square meters for $\Theta^{0}$, $K_{⊥}^{0}$ and $K_{33}^{0}$. They respectively denote the thermal characteristic length, the viscous characteristic length in transverse and normal directions, the static thermal permeability, and finally the static viscous Darcy [27] permeability in transverse and normal directions. However, the open porosity ϕ and high-frequency limit of tortuosity $\tau_{jj}^{\infty }$ are dimensionless and independent of the characteristic length.

### 4.2. Macro-Modulation of Effective Properties

The fluid layer $\Omega_{e}$ is split in N−1 intervals, bounded by *N* linearly placed nodes along $e_{3}$. At $\Gamma_{0}$ there is the first node $x_{3}^{\left(1\right)}=0$, and at $\Gamma_{L}$ we place the *N*-th node at $x_{3}^{\left(N\right)}=L$. Between them, we place the other nodes at $x_{3}^{\left(i\right)}=\left(i-1\right)L/\left(N-1\right)\;,\forall i\in ⟦1,N-1⟧$. The interpolation of the graded properties based on this discretization is done using PCHIP [26]. This ensures the values at the nodes $W_{G}\left(x_{3}^{\left(i\right)}\right)$ and the interpolated function is of class $C^{1}$, meaning that its derivative remains continuous. On the interval $\left[x_{3}^{\left(i\right)},x_{3}^{\left(i+1\right)}\right]$ between two nodes, each geometric property *g* (components of $W_{G}$) reads,

(13) $$\forall x_{3}\in \left[x_{3}^{\left(i\right)},x_{3}^{\left(i+1\right)}\right]\;,x_{3}^{\left(i\right)}<x_{3}^{\left(i+1\right)}\;,\;W_{g}\left(x_{3}\right):=\sum_{n=0}^{3}a_{n,g}{\left(x_{3}-x_{3}^{\left(i\right)}\right)}^{n}\;,$$

with $a_{n,g}$ being the weights of the polynomial interpolation function. It is important for the reader to note that two different interpolations are taking place in the procedure. The first one described in the previous subsection, to interpolate the database of unit-cells, and a second one applied between spatial nodes to interpolate the graded properties of the medium $\Omega_{e}$.

### 4.3. Optimization of Geometric Properties

In order to optimize the geometric parameters at the nodes $W_{G}\left(x_{3}^{\left(i\right)}\right)$, the Nelder–Mead (or downhill simplex) method was used. It is an iterative scheme that minimizes a non-linear cost function, based on a geometric approach [28,29]. From an initial set of geometric nodes $W_{G}\left(x_{3}^{\left(i\right)}\right)$, the method takes a series of steps that tend to minimize the cost function. At each iteration of the method, the set of parameters from the previous iteration is transformed within the space of parameters by means of a simplex. The scheme stops when the convergence is reached (with respect to absolute tolerances) or when the maximum iteration number is attained. The stopping condition is given when the iteration number exceeds $n=2\times 10^{3}$, or when both variations of the cost function $\partial_{n}J$ and of the solution vector $\partial_{n}W_{G}$ do not differ by $1\times 10^{-6}$ between two iterations. The optimization is performed using the function fminsearch within MATLAB^®^2016b. This optimization procedure is bounded so that, as the method scans the space of parameters, the simplex can only exist within the parameter range of the database. The characteristic length was chosen between $\ell_{c}=0.3$ mm and $\ell_{c}=1.5$ mm, and the stretching factor between χ=1 and χ=10. In addition, the open porosity was bounded due to the cell topology, its upper and lower bounds can be derived analytically. The components of $W_{G}$ must therefore satisfy the following constraints,

(14) $$\left\{\begin{array}{c} \phi \in \left[0.56,0.89\right]\;,\\ \chi \in \left[1,10\right]\;,\\ \ell_{c}\in \left[300,1500\right]\times 10^{-6}\;m\;. \end{array}\right.$$

The initial guess used to start the optimization procedure is formed of random geometric parameters, according to a uniform distribution within the proposed bounds. The routine also has to be run multiple times with different initial guesses, to avoid local minima. On the one hand, for the optimization of the non-graded porous material case, where the vector of geometric parameters $W_{G}$ is constant along $e_{3}$, no interpolation is needed and the acoustic propagation problem can be solved exactly using a single layer by the TMM. On the other hand, when the properties are graded along $e_{3}$, it is performed using an interpolation over N=5 nodes, and solved with 40 layers using again the TMM. For each layer, the interpolated database connects $W_{G}\left(x_{3}\right)$ and $W_{J}\left(x_{3}\right)$, as to reach $\rho_{\left(e\right)}\left(x_{3},\omega \right)$ and $B_{\left(e\right)}\left(x_{3},\omega \right)$. This way, the acoustic features of the graded and non-graded materials are driven by the geometric properties of the porous structures.

Two distinct cost functions are introduced. The first one is based on the diffuse field absorption,

(15) $$J_{dif}:=\frac{1}{N_{f}}\sum_{i=1}^{N_{f}}{\left(\alpha_{dif}\left(f_{i}\right)-1\right)}^{2}\;,$$

while the second one is based on the arithmetic average of the absorption,

(16) $$\bar{J}:=\frac{1}{N_{f}}\sum_{i=1}^{N_{f}}{\left(\bar{\alpha }\left(f_{i}\right)-1\right)}^{2}\;,$$

with $f_{min}$ and $f_{max}$ the lower and upper frequency bounds, respectively. In the frequency domain, the minimization operates on $N_{f}$ linearly spaced values so that $\Delta f=\left(f_{max}-f_{min}\right)/\left(N_{f}-1\right)$. Both the diffuse field absorption Equation (11) and the average absorption Equation (12) are studied, using a linear discretization over the elevation angle $\theta \in \left[\pi /30,\pi /2\right]$ with $N_{\theta }=30$ points. It gives Δθ=(π/2−π/30)/30≈0.06 rad as the angular elevation step over all possible incidences.

Considering each cost function, the algorithm will maximize the absorption within targets of frequency and elevation angle. The vector of geometric parameters $W_{G}$ is optimized to create graded and non-graded materials. The database that links the geometric parameters $W_{G}$ with the JCAL parameters $W_{J}$ is used to obtain Equation (5). This equation is finally solved using TMM as it is usually faster, and provides the absorption coefficient for the graded and non-graded materials.

## 5. Results

Results of the optimization procedure are shown for different acoustic targets, in order to highlight the benefits of anisotropic and graded effective properties with respect to the isotropic and non-graded ones. We first display the results in the case of a single frequency, when the porous layer operates in its sub–wavelength regime, that is, when the wavelength of the incident wave was larger than the thickness of the porous material ($\lambda =c_{0}/f>L$). In this work, we consider the length L=25 mm. Then, the optimization was applied to broadband acoustic absorption, on the frequency range between 1 kHz and 5 kHz, in other words, λ/L=13.6 and λ/L=2.7 respectively.

### 5.1. Sub–Wavelength Acoustic Absorption

We consider a single frequency f=1000 Hz at which the monochromatic plane wave impinges the porous layer either with anisotropic or isotropic properties. The cost functions described earlier in Equations (15) and (16) do not need the sum over frequency for this optimization.

**Figure 4:**
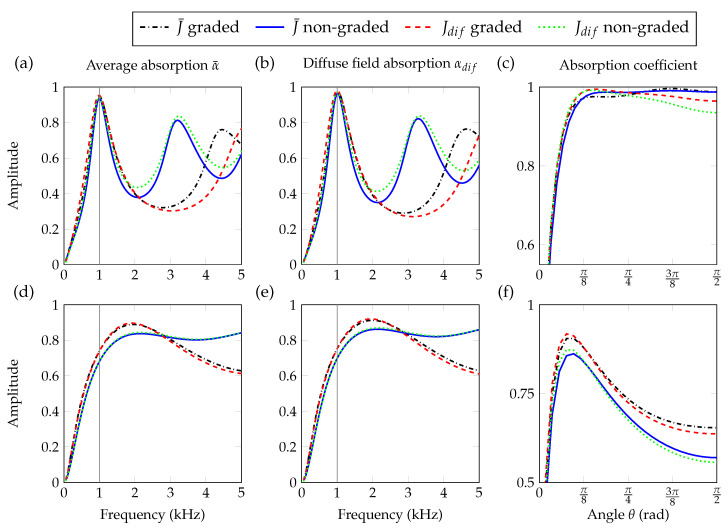
(Color online) Comparison of different optimization results for average and diffuse field absorption coefficients. The angular dependence of the absorption coefficient is given at the frequency f=1000 Hz, with respect to θ. Results with anisotropic cells are given in (**a**–**c**), while the restriction to isotropic cells is shown in (**d**–**f**). In solid red and dashed blue are the results for $\bar{J}$ with N=1 and N=5, respectively, and for $J_{dif}$ with N=1 in dashdotted green and N=5 in dotted black.

Figure 4a,b,d,e show the average absorption coefficients and the diffuse field absorption coefficients for anisotropic (isotropic) materials with graded and non-graded properties optimized using $J_{dif}$ and $\bar{J}$, respectively. In all cases, we can see that both the average absorption and the diffuse field absorption (see Figure 4a,b,d,e) present highest values for the graded porous materials at the target frequency.

The difference between the absorption of graded and non-graded materials is more pronounced for isotropic than for anisotropic materials. This result is important to highlight the relevance of the additional degree of freedom introduced by the spatially dependent properties. On the other hand, it is important to compare the values of absorption between the anisotropic and the isotropic materials. In that regard, we can clearly see the improvements of the absorption by the anisotropic materials showing the importance of the added degree of freedom by this feature. For both $\bar{J}$ and $J_{dif}$, the non-graded medium provides excellent results in terms of absorption. As seen on Figure 4a,b, the absorption peak at f=1000 Hz reaches $\bar{\alpha }=0.94$ and $\alpha_{dif}=0.97$. Figure 4c,f shows the angular profile of absorption at f=1000 Hz for anisotropic (isotropic) materials with graded and non-graded properties optimized using $J_{dif}$ and $\bar{J}$. These results show one important feature related to the impact of the cost function on the angular absorption profiles. While optimizing the average absorption does not promote any specific angle (the cost function $\bar{J}$ accounts for all incidences equally), optimizing the diffuse field absorption coefficient does not account for the grazing and normal angles of incidence. Looking at the average absorption, we see that it is high at normal incidence, and creates a plateau until it finally decreases after θ≈π/8 rad as shown in Figure 4c. However, from the optimization for the diffuse field absorption, we can see the smaller values at normal and grazing incidences as the other incident angles present higher weight in the optimization. By comparing Figure 4c,f we notice the improvement of the results by using anisotropic materials.

**Figure 5:**
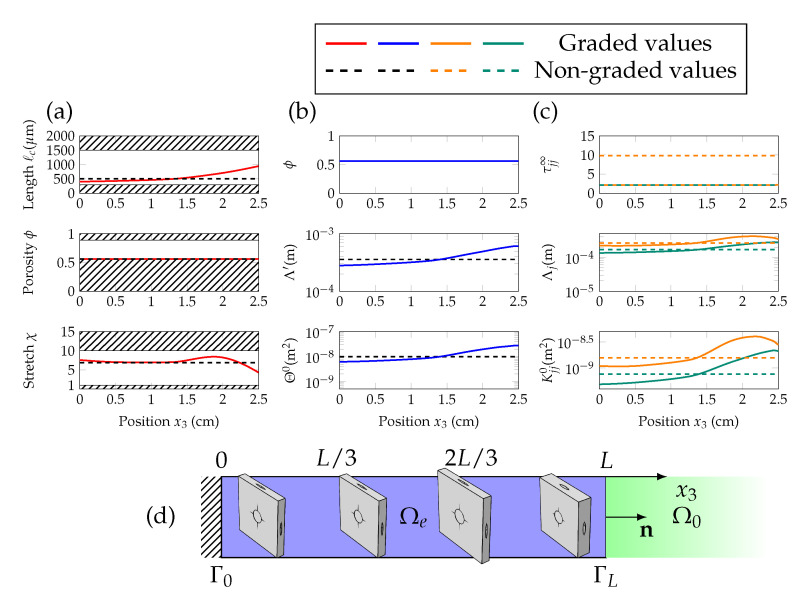
Components of the vector of geometric parameters $W_{g}$ (characteristic length $\ell_{c}$, porosity ϕ and stretch χ) are shown in (**a**), as a function of the position $x_{3}$. Scalar Johnson–Champoux–Allard–Lafarge (JCAL) parameters ($\phi ,\Theta^{0},\Lambda^{\prime }$) are given in (**b**) and the direction-dependent ones ($\tau_{⊥}^{\infty },\tau_{33}^{\infty },K_{⊥}^{0},K_{33}^{0},\Lambda_{⊥},\Lambda_{3}$) are in (**c**), with respect to $x_{3}$. The graded values are in solid lines, and the non-graded ones in dashed lines; in orange for the normal direction, and in green for the transverse direction. The sketch in (**d**) displays snapshots of the unit cells at intervals of L/3 between interfaces $\Gamma_{0}$ and $\Gamma_{L}$.

**Figure 6:**
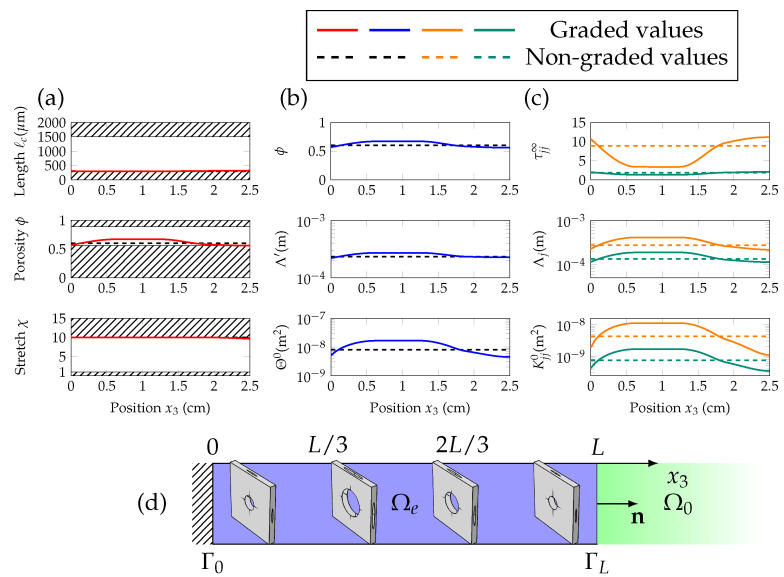
Components of the vector of geometric parameters $W_{g}$ (characteristic length $\ell_{c}$, porosity ϕ and stretch χ) are shown in (**a**), as a function of the position $x_{3}$. Scalar JCAL parameters ($\phi ,\Theta^{0},\Lambda^{\prime }$) are given in (**b**) and the direction-dependent ones ($\tau_{⊥}^{\infty },\tau_{33}^{\infty },K_{⊥}^{0},K_{33}^{0},\Lambda_{⊥},\Lambda_{3}$) are in (**c**), with respect to $x_{3}$. The graded values are in solid lines, and the non-graded ones in dashed lines; in orange for the normal direction, and in green for the transverse direction. The sketch in (**d**) displays snapshots of the unit cells at intervals of L/3 between interfaces $\Gamma_{0}$ and $\Gamma_{L}$.

In the case of anisotropic materials, where the diffuse field and average absorption are strong, grading the properties of the layer along its depth does not help significantly. Looking at the JCAL parameters that give such absorption in Figure 5c and Figure 6c, the medium displays significant anisotropic features. As seen by the high-frequency limit of tortuosity and static viscous permeability, which are one order of magnitude higher in the normal direction $e_{3}$ than in the in-plane ones $e_{⊥}$. Moreover, the snapshots of the unit cells in Figure 5d and Figure 6d, display the shape in the optimized unit cells with large stretch χ, resulting in higher normal tortuosity.

For the sake of comparison, the optimization is also performed with a restriction on the database to isotropic cells, only with χ=1. This restriction implies that the medium cannot take contrasted properties in its principal directions. In this case, using graded properties becomes much more important to achieve a strong absorption, and we reach $\alpha_{dif}=0.76$ and $\bar{\alpha }=0.74$. In Figure 4d,e, the results for an isotropic porous material show the benefit of the geometric gradient $W_{G}\left(x_{3}\right)$, when the medium is constrained to be isotropic.

### 5.2. Broadband Acoustic Absorption

The frequency vector is given by $N_{f}=15$ linearly spaced values between $f_{min}=1000$ Hz and $f_{max}=5000$ Hz. This frequency range ensures the large wavelength condition as we have λ>2L at $f=f_{max}$. This is accounted for in the minimization of the cost functions Equations (15) and (16), by the sum over the frequencies. No weighting is considered in frequency, meaning that all the frequencies have the same importance. The goal of the optimization routine is to minimize the reflection over the whole angular and frequency plane (θ,f), discretized in $N_{f}\times N_{\theta }=450\;$ points.

**Figure 7:**
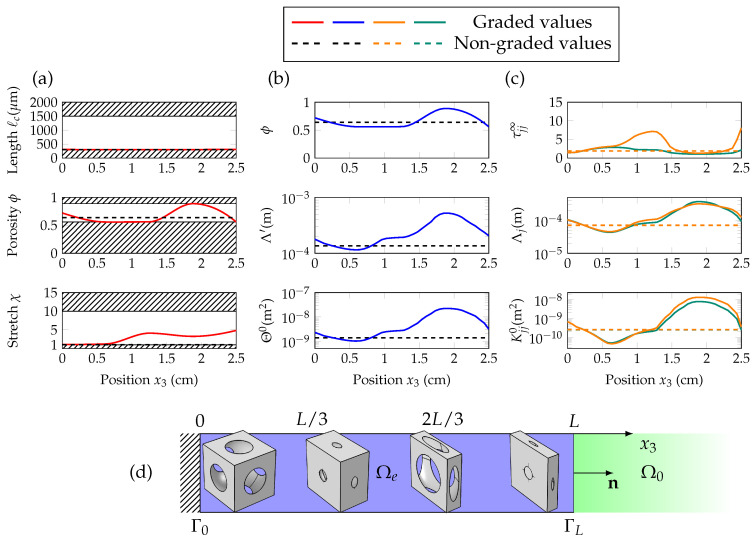
(Color online) Components of the vector of geometric parameters $W_{g}$ (characteristic length $\ell_{c}$, porosity ϕ and stretch χ) are shown in (**a**), as a function of the position $x_{3}$. Scalar JCAL parameters ($\phi ,\Theta^{0},\Lambda^{\prime }$) are given in (**b**) and direction-dependent ones ($\tau_{⊥}^{\infty },\tau_{33}^{\infty },K_{⊥}^{0},K_{33}^{0},\Lambda_{⊥},\Lambda_{3}$) are in (**c**), with respect to $x_{3}$. The graded values are in solid lines, and the non-graded ones in dashed lines; in orange for the normal direction, and in green for the transverse direction. The sketch in (**d**) displays snapshots of the unit cells at intervals of L/3 between interfaces $\Gamma_{0}$ and $\Gamma_{L}$.

**Figure 8:**
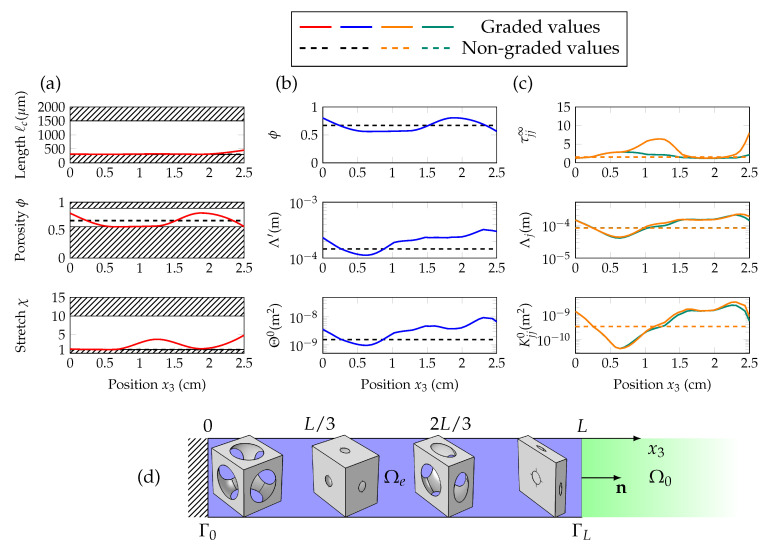
(Color online) Components of the vector of geometric parameters $W_{g}$ (characteristic length $\ell_{c}$, porosity ϕ and stretch χ) are shown in (**a**), as a function of the position $x_{3}$. Scalar JCAL parameters ($\phi ,\Theta^{0},\Lambda^{\prime }$) are given in (**b**) and direction-dependent ones ($\tau_{⊥}^{\infty },\tau_{33}^{\infty },K_{⊥}^{0},K_{33}^{0},\Lambda_{⊥},\Lambda_{3}$) are in (**c**), with respect to $x_{3}$. The graded values are in solid lines, and the non-graded ones in dashed lines; in orange for the normal direction, and in green for the transverse direction. The sketch in (**d**) displays snapshots of the unit cells at intervals of L/3 between interfaces $\Gamma_{0}$ and $\Gamma_{L}$.

Regarding the geometric and JCAL profiles shown in Figure 7a and Figure 8a, we see that the optimized non-graded material is isotropic: χ=1. In this case, over the frequency range of interest, the high-frequency limit of normal tortuosity $\tau_{33}^{\infty }$ does not need to reach important values, for the absorption to reach α>0.9. Figure 7c and Figure 8c show the successive troughs and peaks of the normal tortuosity, when the medium is graded along $x_{3}$. This trend is observed when minimizing for both cost functions, and is intuitively represented on Figure 7d and Figure 8d, as smaller pores and higher transverse stretches tend to increase $\tau_{33}^{\infty }$.

**Figure 9:**
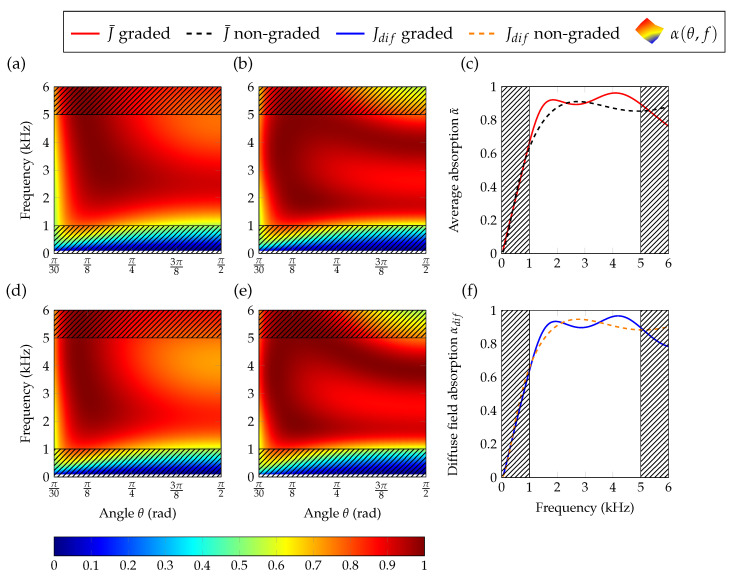
(Color online) Absorption maps with respect to elevation angle and frequency for broadband optimization. The results for optimized average absorption, both non-graded and graded, are shown respectively in (**a**,**b**). The maps (**d**,**e**) display the absorption map for optimized diffuse field absorption coefficient, respectively for non-graded and graded medium. Average absorption is given in (**c**), for graded and non-graded cases in solid red and dashed black respectively. The diffuse field absorption coefficient is shown in (**f**), for graded and non-graded cases in solid blue and dashed orange respectively.

Figure 9a,b shows the average absorption in the plane (θ,f), for the materials made of the optimal profiles with non-graded and a graded properties in the target frequency range minimizing $\bar{J}$. In the same manner, Figure 9d,e shows the diffuse field absorption in the plane (θ,f) for the materials made of the optimal profiles of a non-graded and a graded material in the target range of frequencies minimizing $J_{dif}$ respectively. The optimized non-graded medium provides a large absorption area in the (θ,f) plane, showing a single peak of absorption in the $\left[f_{min},f_{max}\right]$ interval (see Figure 9a,d). However, in the case of the graded material we can clearly observe two peaks of absorption in Figure 9b,e.

The optimized curves of average and diffuse field absorption for a graded material, Figure 9c,f, also clearly show this second peak of absorption for the graded materials in the $\left[f_{min},f_{max}\right]$ interval that is absent in the case of non-graded materials. The maximum of absorption provided by the non-graded medium is at f=(2750±25) Hz with values $\bar{\alpha }=0.91$ and $\alpha_{dif}=0.94$. In the case of graded materials optimized for the diffuse field, the absorption coefficient reaches $\alpha_{dif}=0.93$ at the first peak at f=(1900±25) Hz and $\alpha_{dif}=0.96$ at the second peak f=(4150±25) Hz, while when optimizing the average absorption, the peaks appear at $\bar{\alpha }=0.92$ with f=(1850±25) Hz and $\bar{\alpha }=0.96$ at f=(4100±25) Hz.

This clearly shows the relevance of the spatial profiles of the material that can be used to manipulate the vector of geometric properties $W_{G}\left(x_{3}\right)$ introducing a direct effect on the JCAL parameters $W_{J}\left(x_{3}\right)$ and influencing directly the absorption features of the system. Grading the effective properties, namely the tensor of mass density $\rho_{\left(e\right)}\left(x_{3}\right)$ and bulk modulus $B_{\left(e\right)}\left(x_{3}\right)$, offers more control on the absorption. However, by comparing Figure 9a,d or Figure 9b,e, we can see that for broadband and angular optimization the absorption coefficient displays only small variations depending on the cost function being minimized. Finally, it is worth noting that the peak of the non-graded materials or the double peak for the graded material are still present, with the average of the diffuse absorption coefficients calculated in Figure 9c,f.

At this stage, it is worth analyzing the presence of the second resonance peak in the anisotropic graded material. In order to do so, we represent the reflection coefficient in the complex frequency plane. This methodology has been widely used in the past to gain more insight into the absorption properties of acoustic materials [30,31]. In the absence of losses, this representation presents a unitary reflection in the real frequency axis, due to the conservation of energy, its resonance implies a pair of zero and poles, at complex conjugated frequencies due to the temporal invariance of the system. When losses are introduced in the system, the zeros of the reflection coefficient approach the real axis, fulfilling the impedance match condition (or the critical coupling condition) when the zero is located on the real frequency axis, producing perfect absorption. This technique has been previously described in [14] for the optimization of graded layers at normal incidence, or in [32,33] to explain how to manipulate the zeros of the reflection coefficient in order to achieve perfect absorption.

**Figure 10:**
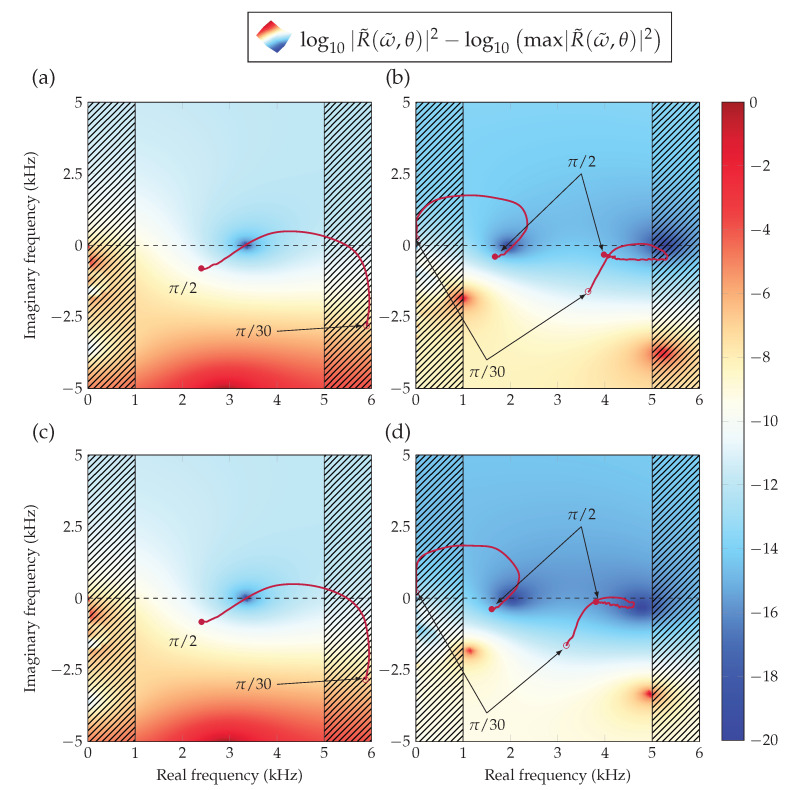
(Color online) Complex frequency plane representation of the reflection coefficient. The amplitude is normalized and reads $\log_{10}{\left|\tilde{R}\left(\tilde{\omega },\theta \right)\right|}^{2}-\log_{10}\left(\max|\tilde{R}\left(\tilde{\omega },\theta \right){|}^{2}\right)$ as a function of $f_{R}$ and $f_{I}$. The maps are plotted at the incidence angle for which the first zero is on the real frequency axis in all optimization cases, ${\bar{J}}^{non-grad}$ (**a**), ${\bar{J}}^{grad}$ (**b**) and $J_{dif}^{non-grad}$ (**c**), $J_{dif}^{grad}$ (**d**). The paths of the complex zeros of reflection are displayed in solid purple, between $\theta_{min}=\pi /30$ (∘) and $\theta_{max}=\pi /2$ (•).

We define the complex-valued angular frequency by $\tilde{\omega }=2\pi \left(f_{R}+i\;f_{I}\right)$, with, $f_{R}\in \left[0,f_{max}\right]\;,f_{I}\in \left[-f_{max},f_{max}\right]$. The differential system of equations in Equation (6) to be solved now reads,

(17) $$\frac{d}{dx_{3}}\left\{\begin{array}{c} p\\ v_{3} \end{array}\right\}-i\;\tilde{\omega }\left[\begin{array}{cc} 0 & {\tilde{\rho }}_{3}\left(\tilde{\omega }\right)\\ {\tilde{B}}_{eq}^{-1}\left(\tilde{\omega }\right) & 0 \end{array}\right]\left\{\begin{array}{c} p\\ v_{3} \end{array}\right\}=0\;,$$

where ${\tilde{B}}_{eq}$ and ${\tilde{\rho }}_{3}$, are respectively the equivalent bulk modulus and equivalent mass density in the normal direction, at complex angular frequency $\tilde{\omega }$. The reflection coefficient $\tilde{R}\left(\tilde{\omega }\right)\in C$ is computed and represented in the complex frequency plane. The perfect absorption α=1 is attained when the zero of complex reflection coefficient is exactly located on the real frequency axis $f_{R}$ so $f_{I}=0$. Therefore, we track the trajectory of the zeros for the non-graded and graded configurations, in terms of the angle of incidence θ. The angle at which the zero will be on the real frequency axis will give the angle of incidence at which the system produces perfect absorption. Figure 10a,c show the complex frequency map of $\tilde{R}$, for the non-graded optimized system at the incidence angle θ, for which the zero is on the real frequency axis, that is, at which perfect absorption is obtained by using $\bar{J}$ and $J_{dif}$, respectively. The trajectory of the zero crosses twice the real frequency axis, showing that two configurations present perfect absorption, but only one stands in the target range of frequencies. Figure 10b,d show the complex frequency map of $\tilde{R}$ for the graded optimized system at the incidence angle at which the first zero is on the real frequency axis, that is, the perfect absorption of the first peak by using $\bar{J}$ and $J_{dif}$ respectively. Here, we can observe two zeros corresponding to the previous two peaks of absorption for the graded case. The trajectories of the zeros cross twice the real frequency axis, showing that there are four configurations with perfect absorption but only two are in the target range of frequencies. There is a general tendency of the zeros independently of the type of material used. In the frequency range of interest, we observe the zeros slowly drifting to higher frequencies as the angle of incidence decreases, visible on Figure 9a,b,d,e.

In contrast to the non-graded materials, the graded ones contribute to lowering the second reflection zero in the frequency range of interest, increasing the absorption across the entire range of frequencies, as shown in Figure 10. Effectively, the broadband absorption is enhanced relative to a strictly non-graded material. These results are in agreement with the previous discussion of the Figure 9c,f.

## 6. Conclusions

In this work, an optimization technique is developed and applied to anisotropic and graded structures. Acoustic wave absorption can therefore be fine-tuned in porous materials, and designed for specific angular and frequency ranges. The proposed optimization routine relies on a database of 100 anisotropic porous unit cells, and provides the physical properties of the desired porous material presenting target absorption features. The effective properties, defined by the mass density tensor and bulk modulus, are graded, and thus macro-modulated along the depth of the porous material. Instead of running the minimization for all JCAL parameters of the equivalent fluid, the geometric parameters are preferred. The average and diffuse field absorption coefficients are considered, and lead to different geometric profiles for rigidly backed layers of finite thickness. While the optimization of the diffuse field absorption coefficient is weighted angularly, reducing the effects of the absorption at normal and grazing incidences, we have seen that using the average absorption can be useful to obtain larger values of absorption at the normal and grazing angles.

Sub-wavelength and broadband absorption objective functions are considered between 1 kHz and 5 kHz for a fixed thickness of 25 mm. From numerical results emerge the benefits of anisotropic and graded effective properties. They are visualized through the use of both the angular and the complex frequency maps, that reveal how the absorption coefficient is maximized. The resulting materials tend to approach omnidirectional absorption, by exploiting the database of anisotropic cells and graded properties. Moreover, as it has previously been found for normal incidence, the graded materials can strengthen the absorption properties by making use of additional resonances, produced by the graded properties of the system.

However, such a procedure is non-exhaustive, since a choice of unit cell and geometric parameters should first be made. It leads to optimized non-unique solutions for a given micro-structure.

Although the present numerical investigation gives promising results, the main hurdle resides in manufacturing such micro-structures. The manufacturing techniques can be difficult to apply for such designs, but the strength of the proposed method is its use on the database. Recent printing techniques can be put into use, but remain limited in terms of surface roughness and overall accuracy at the microscopic scale. In this way, the community is moving towards characterization and imaging techniques, so that surface roughness, and overall discrepancies of the manufactured medium can hence be accounted for, and be quantitatively estimated.

## Abbreviations

The following abbreviations are used in this manuscript:

TMM	Transfer matrix method
FEM	Finite elements method
PS	Peano series
WSTGF	Wave-splitting transfer Green functions
PCHIP	Piecewise cubic Hermite interpolating polynomial
JCAL	Johnson–Champoux–Allard–Lafarge
